# Dissolution of Silk Fibroin in Mixtures of Ionic Liquids and Dimethyl Sulfoxide: On the Relative Importance of Temperature and Binary Solvent Composition

**DOI:** 10.3390/polym14010013

**Published:** 2021-12-21

**Authors:** Omar A. El Seoud, Marc Kostag, Shirley Possidonio, Marcella T. Dignani, Paulo A. R. Pires, Matheus C. Lourenço

**Affiliations:** 1 Institute of Chemistry, The University of São Paulo, Sao Paulo 05508-000, Brazil; k05marc85@gmail.com (M.K.); marcelladignani2@gmail.com (M.T.D.); parpires@iq.usp.br (P.A.R.P.); mrmcosta@hotmail.com (M.C.L.); 2 Department of Chemistry, Institute of Environmental, Chemical, and Pharmaceutical Sciences, Federal University of São Paulo, Sao Paulo 04021-001, Brazil; possidonio@unifesp.br

**Keywords:** silk fibroin dissolution, effects of temperature, ionic liquid molecular structure, design of experiments, molecular dynamics simulations

## Abstract

We studied the dependence of dissolution of silk fibroin (SF) in mixtures of DMSO with ionic liquids (ILs) on the temperature (*T* = 40 to 80 °C) and DMSO mole fraction (*χ*_DMSO_ = 0.5 to 0.9). The ILs included BuMeImAcO, C_3_OMeImAcO, AlBzMe_2_NAcO, and Bu_4_NAcO; see the names and structures below. We used design of experiments (DOE) to determine the dependence of mass fraction of dissolved SF (SF-m%) on *T* and *χ*_DMSO_. We successfully employed a second-order polynomial to fit the biopolymer dissolution data. The resulting regression coefficients showed that the dissolution of SF in BuMeImAcO-DMSO and C_3_OMeImAcO-DMSO is more sensitive to variation of *T* than of *χ*_DMSO_; the inverse is observed for the quaternary ammonium ILs. Using BuMeImAcO, AlBzMe_2_NAcO, and molecular dynamics simulations, we attribute the difference in IL efficiency to stronger SF-IL hydrogen bonding with the former IL, which is coupled with the difference in the molecular volumes and the rigidity of the phenyl ring of the latter IL. The order of SF dissolution is BuMeImAcO-DMSO > C_3_OMeImAcO-DMSO; this was attributed to the formation of intramolecular H-bonding between the ether oxygen in the side chain of the latter IL and the relatively acidic hydrogens of the imidazolium cation. Using DOE, we were able to predict values of SF-m%; this is satisfactory and important because it results in economy of labor, time, and material.

## 1. Introduction

**Figure 1:**
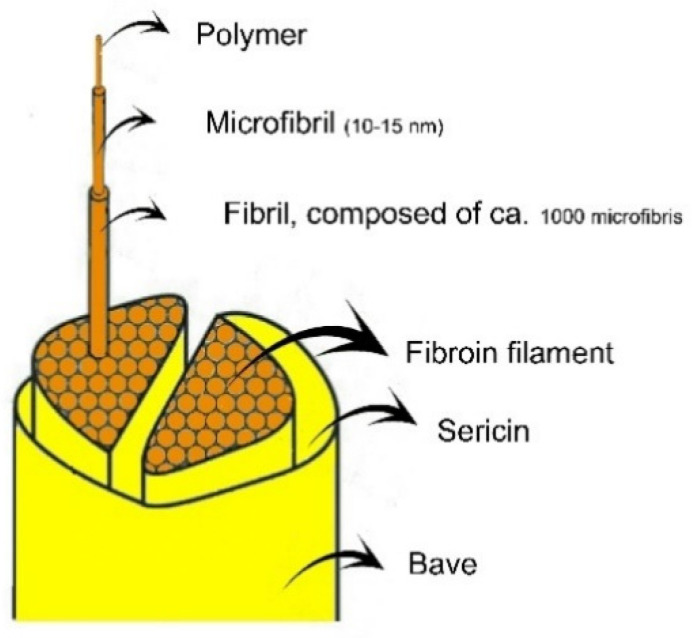
Schematic representation of the structural components of a raw silk fiber from the *B. mori* silkworm.

Natural silk fiber, e.g., that secreted by the domesticated silk worm species *Bombyx mori* (*B. mori*), is composed of two concentric filaments, in addition to small amounts of pigment, wax, and carbohydrates; see Figure 1. The outer filament (silk sericin) is a sticky, hydrophilic glycoprotein that represents ca. 20–30% of the structure of the cocoon depending on environmental/growing conditions [1]. This (water-soluble) layer is usually removed during the industrial processing of silk fibers in a step called “degumming”, which is usually done by heating the cocoons with hot water under pressure or by boiling them in an alkaline (Na_2_CO_3_), soap, or synthetic detergent solution [2]. Sericin removal is required to allow continuous and smooth filament reeling and to achieve the desired luster and touch in silk textiles [3]. The inner part of the fiber is silk fibroin (SF), which is composed of light (L) and heavy (H) polypeptide chains linked at the C-terminus by a disulfide bond. The L-H complex binds a glycoprotein P25 chain in a 6:1 ratio. The H-chains of SF contain twelve hydrophobic, crystalline domains where the amino acids are present in a repetitive sequence composed essentially of glycine (≈43–46%), alanine (≈25–30%), and serine (≈12%), along with some tyrosine (≈5%), valine, and threonine. Additionally, there are eleven hydrophilic, non-crystalline regions where the amino acids are present in a non-repetitive sequence of glutamic, aspartic acid, arginine, and lysine [4].

The SF fiber is assembled from nanofibrils 3–5 nm in diameter that are considered the building block of silk. These twisted bundles of nanofibrils interact strongly with each other, mainly by hydrogen-bonding (H-bonding) and van der Waals interactions [5,6], to form microfibrils with a diameter of 20 to 200 nm. This fibrillar assembly is thought to be responsible for the excellent mechanical strength of silk fibers [7].

Although SF is insoluble in water and many molecular solvents (strongly dipolar aprotic solvents, e.g., dimethyl sulfoxide, DMSO, *N,N*-dimethylacetamide, DMAc, and hexafluoro-2-propanol are exceptions) [8], there is an intense interest in this dissolution because of the potential applications of the regenerated biopolymer, either pure or as nanocomposites especially with cellulose (Cel) in tissue engineering and drug delivery [9]. This dissolution requires disruption of the H-bonds and van der Waals interactions present; this explains the solubility of SF in concentrated solutions of electrolytes that attenuate the above-mentioned interactions. For example, SF is soluble in 9.3 M aqueous LiBr solution (80.76 wt% LiBr!), ethanolic CaCl_2_ solution, as well as electrolyte solutions (e.g., LiCl, SrCl_2_, and ZnCl_2_) in formic acid. Other solvents that dissolve SF include aqueous solutions of strong bases, e.g., choline-, and tetra (*n*-butyl) ammonium hydroxide [10]; aqueous *N*-methyl-*N*-morpholine oxide, and electrolyte solutions in dipolar aprotic solvents e.g., LiCl/DMAc [11]. Finally, solvents that are composed solely of ions, in particular ionic liquids (ILs) and deep eutectic solvents also dissolve SF [9].

**Figure 2:**
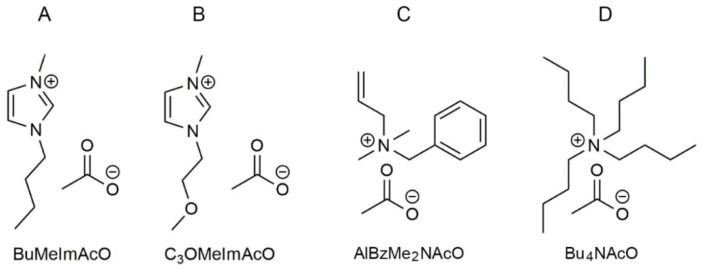
Molecular structure of the ionic liquids employed in the present work: 1-(*n*-butyl)-3-methylimidazolium acetate (BuMeImAcO) (**A**), 1-(2-methoxyethyl)-3-methylimidazolium acetate, (C**_3_**OMeImAcO) (**B**), allylbenzyldimethylammonium acetate (AlBzMe**_2_**NAcO) (**C**), and tetra(*n*-butyl) ammonium acetate (Bu**_4_**NAcO) (**D**).

The present work is on the dissolution of SF in the ILs shown in Figure 2; these are solvents composed only of ions and have melting points, by operational definition, ≤100 °C. The ILs that we investigated have the same anion (acetate); the cations are derivatives of imidazole, 1-(*n*-butyl)-3-methylimidazolium acetate (BuMeImAcO), 1-(2-methoxyethyl)-3-methylimidazolium acetate, (C**_3_**OMeImAcO), and quaternary ammonium ions, allylbenzyldimethyl ammonium acetate (AlBzMe**_2_**NAcO), and tetra(*n*-butyl) ammonium acetate (Bu**_4_**NAcO). Binary mixtures of these ILs with DMSO dissolve Cel [12]. The use of a co-solvent (DMSO) for Cel dissolution is advantageous because it reduces the biopolymer solution viscosity, leading to better heat and mass transfer [13]. The effects of the dissolution temperature (*T*) and the composition of the binary solvent mixture (given by *χ*_DMSO_, the mole fraction of DMSO) on the dissolution of Cel were assessed using chemometrics (design of experiments, DOE). Cel dissolution increased as a function of increasing both variables; the contribution of *χ*_DMSO_ was larger than that of *T* for some ILs [14].

Note that the literature on SF dissolution and on elucidation of the biopolymer dissolution mechanism is scarce. To the best of our knowledge, there are only few studies on the dissolution of SF in ILs; the emphasis in these studies was on the dependence of biopolymer dissolution, which was given as mass%, SF-m%, on the molecular structure of the cation and anion of the IL [15,16,17,18,19]. Using mixtures of DMSO with the ILs shown in Figure 2, we carried out the present study in order to *assess the relative importance to SF-m% of T and χ_DMSO_*. We were interested in probing the effect on SF-m% of the presence of a Lewis base (an ether linkage) in the side chain of C**_3_**OMeImAcO as compared with BuMeImAcO. The quaternary ammonium ILs differ in the volumes of the attached groups; the phenyl ring of AlBzMe**_2_**NAcO is rigid, unlike the flexible *n*-butyl groups of Bu_4_NAcO; both factors may affect SF-m%.

Using the DOE statistical approach, we assessed the relative importance of the above-mentioned experimental variables. Interestingly, we found that SF-m% depends more on *T* than on *χ*_DMSO_ for BuMeImAcO and C_3_OMeImAcO; the inverse is true for AlBzMe**_2_**NAcO and Bu_4_NAcO. Thus, for this limited group of ILs, our quantitative results highlight the experimental variable that should be “modulated” in order to enhance biopolymer dissolution. We also calculated values of SF-m% under conditions *other than those employed to generate the statistical model* and determined the corresponding SF-m% experimentally. The excellent agreement between both values (3.8 ± 2%) shows the robustness of the statistical model, and the usefulness of our approach to predict biopolymer dissolution, thus saving time, labor, and material.

## 2. Materials and Methods

### 2.1. Materials

The reagents and solvents were purchased from Sigma-Aldrich (Milwaukee, WI, USA) and Acros Organics (Geel, Belgium) and were purified as given elsewhere [20]. Silk was a commercial degummed sample from Bratec (Londrina, PR, Brazil). The solvatochromic dye 2,6-dichloro-4-(2,4,6-triphenylpyridinium-1-yl)phenolate (WB) was available from a previous study [21].

### 2.2. Methods

#### 2.2.1. Further Degumming of Silk Fibroin

We carried out this step according to references [22,23] by agitating 100 g of the silk sample with 1 L of aqueous sodium dodecylbenzene sulfonate (2 wt%) at 80 °C for 1 h, which was followed by fiber filtration. We subjected the resulting fibers to the following sequence: (i) agitation with 1 L of water (60 °C) and filtration; (ii) agitation with 500 mL of ethanol (50 °C) and filtration; (iii) drying in air, and then under reduced pressure at 60 °C (oven), over P_4_O_10_ until constant mass. Steps (i) and (ii) were repeated three times each.

#### 2.2.2. Characterization of the Degummed SF

##### Fiber Morphology by Scanning Electron Microscopy (SEM)

We show below the SEM micrographs for the commercial SF (as received), that after further degumming, and of a sample dissolved and then regenerated (in water) from BuMeImAcO-DMSO binary mixture. We used the following steps. We dried the regenerated SF sample, sputtered it with platinum using Baltec model MED-020 coating system (Balzers, Liechtenstein) and recorded the images with LEO Stereoscan 440 electron microscope (Beaver Falls, PA, USA), using high vacuum mode with 20 kV and a secondary electron detector.

##### Fiber Index of Crystallinity Index (I_c_) by FTIR

To ensure a homogeneous covering of the KBr powder with SF in the FTIR experiment, we covered a mixture of ca. 1.5 mg of SF and ca. 150 mg freshly dried KBr with dry 2-propanol. After the suspension was ground thoroughly using a mortar and pestle, we evaporated the solvent under reduced pressure, pressed the powder sample into a pellet, and then recorded the spectrum using a Bruker Vector 22 FTIR spectrophotometer (Karlsruhe, Germany; 64 scans; 1 cm^−1^ digital resolution). We calculated the value of the index of crystallinity (**I_c_**) from the intensity ratio of the peaks at ca. 1260 and ca. 1235 cm^−1^, using Equation (1): [24]

**I_c_** [%] = (A**_1260_** _cm^−1^_/A**_1235_** _cm^−1^_) × 100 (1)

where A**_1260_** and A**_1235_**refer to the absorbances at ca. 1260 cm^−1^ and at ca. 1235 cm^−1^, respectively.

##### Determination of Molar Mass (M_v_) of SF by Viscosity Measurement

We evaluated the rheological properties of the SF solutions (2–6% *w/v*) in 9.3 M LiBr using a Brookfield RS-CPS plus Rheometer (Middleboro, MA, USA), 50 mm diameter cone, and plate geometry with 1° cone angle at 25 °C, over a shear rate range of 0.6 to 1000 s^−1^. We calculated the viscometric molar mass (M**_v_**) of SF using Equation (2) and the Mark–Houwink–Sakurada coefficient (MHS) reported elsewhere [25]:

η**_int_** = KM**_v_^α^** (2)

where η**_int_** is the solution intrinsic viscosity; K is a constant that depends on the polymer and the solvent at a given temperature; and **α** is the MHS exponent. We employed the values given elsewhere for SF, namely K = 1.813 × 10**^−4^** L/g, and α = 0.614, which were calculated using data of size-exclusion chromatography with multiple angle laser light scattering (SEC-MALLS) of SF solution in 0.2 M NaCl [25].

#### 2.2.3. Synthesis of the Ionic Liquids

We synthesized BuMeImAcO as given elsewhere [26] by reacting 17.0 mL of 1-bromobutane (0.158 mol) with 13.2 mL 1-methylimidazole (0.165 mol) in 30 mL of dry ethyl acetate. We stirred the reaction mixture under reflux for 2 h, cooled it, separated the lower phase (IL), and then washed it three times, each with 50 mL of cold ethyl acetate to remove excess 1-methylimidazole; the IL was directly employed in the next step. We performed the transformation BuMeImBr → BuMeImAcO using ion exchange resin (Amberlite IRN 78, 1.20 equivalent OH^−^·mL^−1^). First, we transformed the resin into its acetate form by adding 14.4 mL acetic acid (0.252 mol) to 200 mL of the resin-OH (0.24 mol) suspended in 500 mL of cold water, which was followed by agitation for 1 h. We filtered the resin using a fritted glass column, washed it with water until free of excess acetic acid, and then washed it with methanol. We performed the transformation (BuMeImBr → BuMeImAcO) by slowly passing the BuMeImBr solution in 500 mL methanol over the resin. To verify the completeness of the ion exchange, we added a droplet of the eluted solution to aqueous AgNO**_3_**/HNO**_3_**; no precipitate was observed. After removing methanol, we dried the IL under reduced pressure, over P_4_O_10_ until constant mass, yield of BuMeImAcO = 85%.

^1^H NMR (300 MHz, CDCl_3_, δ in ppm): 10.88 (s, 1H, C2-***H***), 7.38 (d, 1H, C4-***H***), 7.29 (d, 1H, C5-***H***), 4.28 (t, 2H, N-C***H_2_***), 4.05 (s, 3H, N-C***H_3_***), 1.95 (s, 3H, acetate-C***H_3_***), 1.85 (m, 2H, N-CH_2_-C***H_2_***), 1.36 (m, 2H, C***H_2_***-CH_3_), 0.95 (t, 3H, CH**_2_**-C***H_3_***).

We synthesized AlBzMe**_2_**NAcO as given elsewhere [12]. The IL was obtained by reacting allyl bromide with 5 mol% excess *N*-benzyl-*N,N*-dimethylamine, under reflux in acetonitrile (MeCN) for 6 h. The workup of AlBzMe_2_NBr and the transformation (AlBzMe**_2_**NBr → AlBzMe**_2_**NAcO) was similar to that of BuMeImAcO, yield of AlBzMe**_2_**NAcO = 91%.

^1^H NMR (300 MHz, DMSO-*d6*, δ in ppm): 7.60 (d, 2H, benzyl-***H3,5***), 7.49 (m, 3H, benzyl-***H2,4,6***), 6.12 (m, 1H, allyl-C***H***), 5.67–5.62 (m, 2H, allyl-C***H_2_***), 4.56 (m, 2H, benzyl-C***H_2_***), 4.00 (d, 2H, allyl-NC***H_2_***), 2.92 (s, 6H, methyl-C***H_3_***), 1.55 (s, 3H, acetate-C***H_3_***).

We synthesized C**_3_**OMeImAcO by reacting 1-methylimidazole with 1-chloro-2-methoxyethane in MeCN under pressure (10 atm, 6 h, 85 °C), in PTFE-lined stainless-steel reactor. This was followed by removal of the volatiles and product washing with cold ethyl acetate [27]. The produced 1-(2-methoxyethyl)-3-methylimidazolium chloride was transformed into C_3_OMeImAcO using ion exchange, as given for BuMeImAcO, yield of C**_3_**OMeImAcO = 86%.

^1^H NMR (300 MHz, CDCl_3_, δ in ppm): 11.35 (s, 1H, Im C2-***H***), 7.45 (d, 1H, Im C4-***H***), 7.38 (d, 1H, Im C5-***H***), 4.55 (t, 2H, N-C***H_2_***), 4.04 (s, 3H, N-C***H_3_***), 3.74 (t, 2H, O-C***H_2_***), 3.35 (t, 3H, O-C***H_3_***), 1.98 (s, 3H, acetate-C***H_3_***).

We synthesized tetra (*n*-butyl) ammonium acetate (Bu**_4_**NAcO) by adding tri-*n*-butylamine (44.24 g; 0.239 mol) to a solution of 1-bromobutane (39.75 g; 0.290 mol) in 75 mL MeCN. We stirred the mixture under reflux for 48 h, removed the volatiles under reduced pressure, and performed the transformation (Bu**_4_**NBr → Bu**_4_**NAcO) by ion exchange as given for BuMeImAcO, yield of Bu_4_NAcO = 73% [12].

^1^H NMR (300 MHz, CDCl_3_, δ in ppm): 3.35 (t, 8H, N-C***H_2_***), 1.64 (m, 8H, N-CH_2_-C***H_2_***), 1.93 (s, 3H, acetate-C***H_3_***), 1.43 (m, 8H, -C***H_2_***-CH_3_), 1.01 (t, 12H, -C***H_3_***).

#### 2.2.4. Dissolution of Silk Fibroin in IL/DMSO

**Figure 3:**
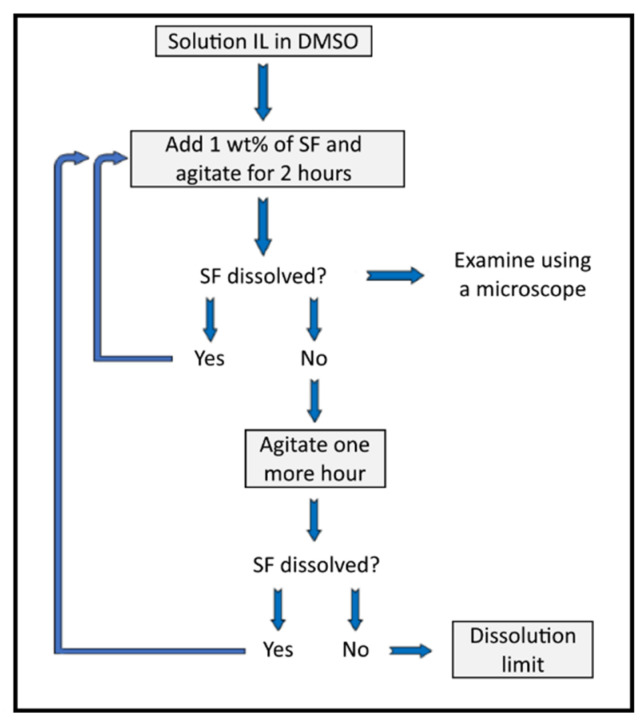
Flow chart for the steps of silk fibroin dissolution in ionic liquid/DMSO binary mixtures, adapted with permission from Dignani et al. [14], adapted with permission, MDPI, 2020.

**Figure 4:**
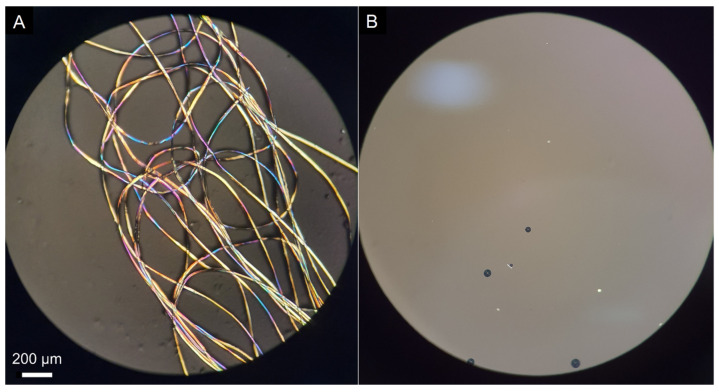
Optical microscope images of undissolved silk fibroin (**A**), and dissolved one (**B**) in AlBzMe_2_NAcO/DMSO.

We used our previously reported dissolution equipment and protocol [28]. A flow chart of the dissolution protocol is depicted in Figure 3. We weighted aliquots of 3–4 g of the IL/DMSO mixtures (*χ*_DMSO_ = 0.5, 0.7, 0.9) into the appropriate glass tubes (borosilicate glass 21 × 60 mm, capacity ca. 15 mL, provided with threaded polybutylene terephthalate screw cap) and then introduced dry SF fibers (*ca*. 1 wt% or 30 mg, of SF fibers of ca. 0.5 cm length) and agitated the suspension at the required temperature (40, 60, 80 °C) for 2 h. The stirring speed was kept at its maximum value (350 rpm; digital laser tachometer, model 2234C+, Signsmeter) as long as the SF solution/suspension does not creep up the cylindrical part of the PTFE agitation blade. When this occurred, usually at high polymer concentrations, the stirring speed was reduced. We judged SF dissolution visually (*without opening the glass tube*) under 12× magnifying glass provided with LED light. We reached the final decision (on dissolution) with the aid of a microscope (Nikon, Eclipse 2000 microscope with cross-polarization); see Figure 4. In case of complete dissolution (dark image under the microscope), we added more SF, namely, 3 wt%, 2 wt%, 1 wt%, or 0.5 wt% increments of the total mass of (IL + DMSO + the SF already added) and repeated the agitation/solution examination sequence. We defined the (operational) dissolution limit when the biopolymer remained undissolved after 3 h from the last addition. We report the solubility of SF in the respective solution as mass percentage, SF-m% = (m_SF_/(m_SF_ + m_IL_ + m_DMSO_) × 100); m = mass. The uncertainty in the maximum dissolved SF was calculated by [(SF-m%)_maximum_ − (SF-m%)_minimum_)/(SF-m%)_maximum_] × 100.

#### 2.2.5. Spectrophotometric Determination of the Empirical Polarity E_T_(WB) of Silk Fibroin Solutions in Ionic Liquid-DMSO Mixtures

We employed WB as a solvatochromic probe to calculate the empirical polarity of the SF/BuMeImAcO-DMSO solutions as a function of solvent composition under the following conditions: final concentration of WB = 5 × 10**^−4^** mol/L.; SF concentration = 4 wt%; *χ*_DMSO_ = 0.5, 0.6, 0.7, 0.8, 0.9; *T* = 40 °C. We employed Shimadzu UV-2550 UV/Vis spectrophotometer (Kyoto, Japan), equipped with a digital thermometer (model 4000A, Yellow Springs Instruments; Yellow Springs, OH, USA) that measured the temperature inside the cell-holder (±0.05 °C). We recorded each spectrum 3 times at a resolution of 0.2 nm and calculated the value of *E***_T_**(WB) from Equation (3), where λ**_max_** refers to the wavelength of maximum absorption of the solvent-sensitive (i.e., solvatochromic) peak of WB. We calculated the values of λ**_max_** from the first derivative of the absorption spectra [29]:

*E*_T_(WB) = 28591.5/λ**_max_**. (3)

#### 2.2.6. Density Measurement of Solutions of Silk Fibroin in Mixtures of Ionic Liquids and DMSO

We used Anton Paar DMA 4500 M digital density meter (Graz, Austria) to measure the densities of SF solutions whose compositions are shown in Table 1 at 60 °C.

#### 2.2.7. Silk Fibroin Dissolution Studied Using Design of Experiments (DOE)

Using the Statistica Software (version 13.0, Dell, USA), we employed DOE [30,31,32] to determine the relationship between SF-m% and *two independent experimental variables*, namely the dissolution temperature *T* and the composition of the binary solvent, which are expressed by the mole fraction of DMSO, *χ*_DMSO_. We tested three values (or levels) for each of these variables: *T* = 40, 60, 80 °C and *χ*_DMSO_ = 0.5, 0.7, 0.9. According to DOE, the number of experiments is 9 (=3^2^). To increase the statistical robustness of the data, we repeated the central point (*T* = 60 °C and *χ*_DMSO_ = 0.7) three more times, giving a total of 12 runs for each IL-DMSO solvent. The order of design points was randomized to reduce the effect of unpredicted variables. Response surfaces (vide infra) were generated by the response surface methodology, RSM as implemented in the Statistica software.

It is customary to designate the levels of the experimental variables by numbers, e.g., the three levels of *T* are designated −1 (=40 °C), 0 (=60 °C), +1 (=80 °C); we employed the same methodology for *χ*_DMSO_. Table S1 (Table S1 of Supplementary Material) shows the (randomized) order of carrying out the SF dissolution experiments for each IL-DMSO binary solvent. We calculated the reduced scales of the variables from Equations (4) and (5):

Reduced T = (T − 40)/(80 − 40)(4)

Reduced *χ*_DMSO_ = (*χ*_DMSO_ − 0.5)/(0.9 − 0.5) (5)

#### 2.2.8. Silk Fibroin Dissolution, Studied by Molecular Dynamics (MD) Simulations

We used the Gromacs 2020.5 software package [33] to simulate two SF/binary solvent systems, each containing the following number of molecules: DMSO, 1750; IL, 750; and a model for spider silk fibroin (thereafter designated as SF crystal). The coordinates of the latter were obtained from the Protein Databank (PDB Id-1slk) [34]. This model is composed of an ensemble of 15 chains of the hexapeptide GAGAGA (G = glycine, A = alanine) sequence. These chains are spatially arranged to form a β-sheet secondary protein structure with a ratio between parallel and antiparallel chains of 1:2. The above-mentioned SF crystals contain, in each chain, an acetyl group in the hexapeptide N-terminal and a metylamine linked to the C-terminal amino acid residue.

We kept this structure and, as indicated elsewhere, we changed the terminal alanine residue of each strand to serine in order to mimic the amino acid sequence observed in *Bombyx mori* silk [35]. Figure S1 shows the molecular structures of the parallel and antiparallel SF chains employed in the MD simulations. We generated the simulation boxes using the PACKMOL program [36] and performed the simulations at 333 °K (60 °C) for 500 ns with a time step of 2 fs by using an OPLS (Optimized Potential for Liquid Simulations) force field for all molecules, isothermal–isobaric (NPT) condition, periodic boundaries, and the smooth particle-mesh Ewald (PME) algorithm for long-range electrostatic interactions [37], with a PME order equal to 4 and Fourier space equal to 0.12. Other simulation details are: cutoff distances (electrostatic and van der Waals) = 1.2 nm, velocity-rescaling thermostat, three reference groups (the SF crystal, DMSO, and IL molecule), with time constant = 0.2 ps; Parrinello–Rahman barostat with isotropic coupling type, time constant = 5 ps, reference pressure = 1 bar. The binary solvent mixture isothermal compressibility was taken equal to that of DMSO, i.e., 5.23 × 10^−5^ bar^−1^. All other simulation parameters are the default ones defined by Gromacs. We checked the equilibration of the ensemble by monitoring the potential energy and solution density as a function of simulation time. We found that the potential energy curves typically reached equilibrium values (i.e., remains essentially constant) after ca. 10 ns simulation time. We optimized the geometries (gas phase) of IL cations (BuMeIm^+^, AlBzMe_2_N^+^) and the acetate ion using the DFT method, with a B3LYP functional and cc-pVDZ basis set, as implemented in Gaussian 09; we calculated the molecular volumes of the IL cations similarly. We generated the topology files of the OPLS force field using MKTOP [38] and calculated the partial charges on the atoms based on the RESP (restrained electrostatic potential fit) approach [39], as calculated by a combination of Gaussian09 and Ambertools 20 suite [40]. We used published data for OPLS-optimized DMSO geometry and topology [41].

For the analysis of MD simulation results, we employed the radial distribution functions (RDF), mindist (minimal distance between one species and the other), root mean square deviations (RMSD), solvent accessible surface area (SASA), and number of H-bonds as implemented in the Gromacs package and VMD 1.9.3 software [42]. The criterion that we employed for counting each H-bond is that the distance between the donor and acceptor atom is ≤0.3 nm, and the angle formed between these atoms is ≤20°.

The final volumes of the simulation boxes were 457.218 and 499.848 nm^3^ for the binary mixtures of DMSO with BuMeImAcO and with AlBzMe_2_NAcO, respectively. We employed solution density to validate the simulation conditions. Table 1 shows the concentrations of the species in the simulation boxes, the experimental densities, and those calculated by MD simulations. We calculated the extension of the first solvation layer of the SF crystal in different IL/DMSO from the minima of the RDF curves, as shown by the arrows in part B of Figure S2. An RDF curve refers to the average density of all atoms of DMSO and IL inside the simulation box as a function of the distance from the ensemble surface.

## 3. Results and Discussion

### 3.1. Physicochemical Characteristics of Silk Fibroin

**Figure 5:**
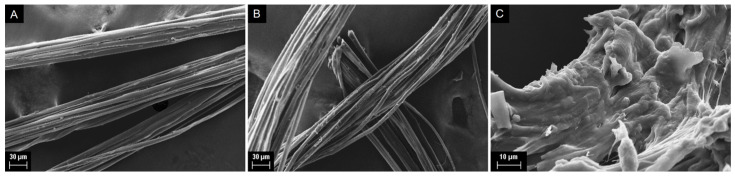
SEM micrographs of silk as received (**A**), after additional degumming (**B**), and that dissolved in 1-butyl-3-methylimidazolium acetate and then regenerated in water (**C**). The micrograph scales are 30 μm (parts (**A**,**B**)) and 10 μm (part (**C**)).

For the degummed SF, we calculated the value of **I_c_** *=* 0.86 from FTIR, as compared with 0.66 [24], 0.72, and 0.84 for Mulberry silk, regenerated SF film [43], and electrospun SF mats, respectively [44]. The average molar mass that we calculated from the viscosity of SF aqueous solutions in LiBr [25], (1154 ± 367) KDa, is higher than those of some SF samples reported in the literature, e.g., from 100 to 250 kDa [45]; 390 kDa [46], and 411 kDa [47]. The SEM micrographs of Figure 5 show clearly that the physical integrity of SF was not impaired by the extra degumming that we carried out (parts A and B of Figure 5). However, part C of Figure 5 shows the profound effect on the fibrous structure of SF when the biopolymer is dissolved in IL-DMSO and then regenerated in water.

### 3.2. Rational for the Choice of the Molecular Structures of the ILs

As shown in Figure 2, all ILs employed are acetates that differ in the molecular structures of the cations; two are derivatives of imidazole, whereas the other two are quaternary ammonium acetates. As in the case of Cel [48,49], biopolymer-solvent H-bonding and van der Waals interactions are determinant for its dissolution. We dwell on H-bonding between SF and the IL cation. The 1,3-disusbtituted imidazolium cation acts as a Lewis acid via its relatively acidic C2-***H*** of the imidazolium ring [50]. Additionally, C_3_OMeImAcO carries an ether link in its side chain that can, *in principle*, donate electrons to the relatively acidic hydrogens of SF, e.g., -CO-N***H***-. The relevance of these structural differences to SF dissolution can be readily assessed by a quantitative study of biopolymer dissolution, *vide infra*. The α-hydrogens of the quaternary ammonium ions of AlBzMe_2_NAcO and Bu_4_NAcO are less acidic than C2-***H*** of the imidazolium ring. Therefore, it is plausible that the contribution of biopolymer-cation H-bonding to SF dissolution is less important than in case of the imidazole-based ILs. Again, this conclusion can be assessed by the quantitative study discussed below.

### 3.3. Quantitative Study of Silk Fibroin Dissolution in Ionic Liquid-DMSO Binary Mixtures: Use of Chemometrics

As given in the Experimental section, we studied the effects of *two independent experimental variables*, *T* and *χ*_DMSO_, on the dissolution of SF. An approach that can be employed is the *one-at-a-time variation*. Thus, we fix one experimental variable, e.g., *T*, and vary *χ*_DMSO_ systematically until (SF-m%)_maximum;DMSO_ is reached. Then, we carry out a second set of experiments using the *χ*_DMSO_ that resulted in (SF-m%)_maximum;DMSO_ at different *T* until a new maximum, (SF-m%)_maximum;DMSO;T_ is reached. Although useful, this protocol does not guarantee *a real* maximum for both variables. However, equally important is that the one-at-a-time approach gives no indication about the relative importance to SF dissolution of each experimental variables. However, chemometrics should be used, where both factors are varied *simultaneously in a random manner*; see Table S1 [30]. Our results are listed in Table 2; they show that we repeated the central point three more times for increasing the robustness of the generated statistical model. The salient feature of Table 2 is that the imidazole-based ILs are more efficient than the quaternary ammonium ILs. A rational for this dependence will be offered below based on our MD data.

Based on the results of Table 2, we tested the fit of mathematical models to the SF dissolution data; the second-order polynomial gave an excellent fit, as evidenced by the values of R**^2^** of Table S2. The latter table shows the equations that we calculated using the raw data for the dependence of SF-m% on *T* and *χ*_DMSO_. According to the second-order polynomial, there are terms in *T* and *T***^2^**, *χ*_DMSO_ and (*χ*_DMSO_)**^2^** and a “mixed” term *T* × *χ*_DMSO_. Since the experimental variables have different scales, we evaluated the relative importance of *T* and *χ*_DMSO_ to the dissolution of SF by using reduced scales; see Equations (4) and (5) of the Experimental section, and we calculated the equations shown in Table 3. We dwell on the first-order terms because the second-order and mixed terms are required for achieving a better statistical fit. As shown from the regression coefficients, the effect of *T* is more important to SF dissolution than *χ*_DMSO_ (entries 1 and 2 of Table 3); the inverse is true for the quaternary ammonium ILs (see entries 3 and 4). We will use our MD results to offer a rational for the difference between the two classes of ILs, vide infra.

**Figure 6:**
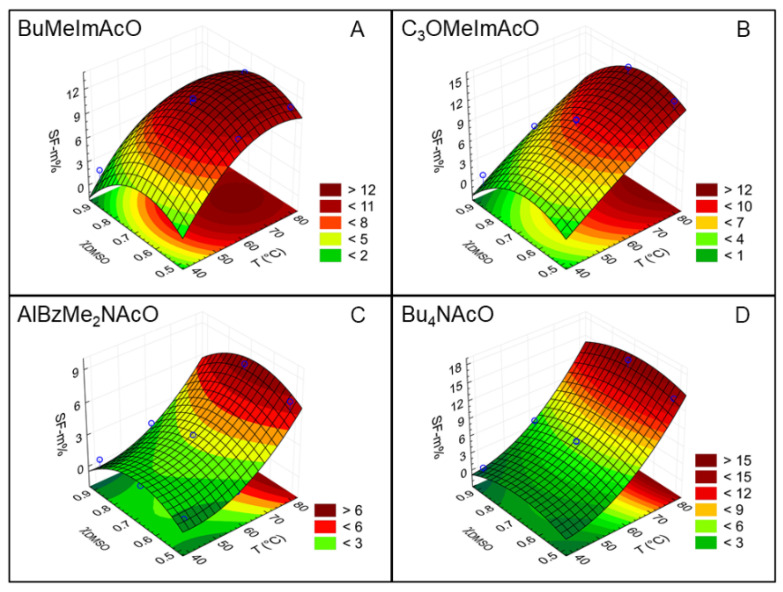
Response-surface plots for the dependence of silk fibroin dissolution on the temperature *T*, and mole fraction of DMSO, *χ*_DMSO_ for the four IL-DMSO mixtures employed. We generated these plots from the data of Table S2, using second-order polynomials. Parts (**A**–**D**) refer to the ionic liquids studied.

The color-coded Figure 6 shows the profiles for the quadratic response surface plots of the optimization of *T* and *χ*_DMSO_. Figure 6A represents a surface where the maximum and minimum points are within the experimental region, Figure 6B shows that the maximum is not far from the experimental region, whereas the remaining parts of Figure 6 represent cases where the maximum is still outside the experimental region [51,52].

We validated the quality of fit of the model by running additional experiments under experimental conditions that are different from those employed to generate the mathematical equations; the results are listed in Table 4. As shown, the model is robust, leading to differences of (3.8 ± 2%) between calculated and experimental values. This predictive power is not only satisfying but, more importantly, it saves time, labor, and material.

As in case of Cel, the solvent empirical polarity *E**_T_***(WB) can, in principle, be employed to correlate SF-m% [48]. However, *E**_T_***(WB) is a dependent variable; i.e., its value is determined by *T* and *χ*_DMSO_. Indeed, *E**_T_***(WB) correlates smoothly with *T* at a fixed *χ*_DMSO_ and with *χ*_DMSO_ at a fixed *T*, as shown in Table S3. Therefore, it is not possible to use *E_T_*(WB) instead of *χ*_DMSO_ or *T* in Table 3, because medium empirical polarity is strongly correlated with the independent variables studied. However, the relevant point is that *there is theoretical and experimental ground for using the E_T_(probe) to assess the efficiency of SF solvents*.

### 3.4. A Molecular Dynamics-Based Rationale for the Dependence of Silk Fibroin Dissolution on the Molecular Structure of the Ionic Liquids

**Figure 7:**
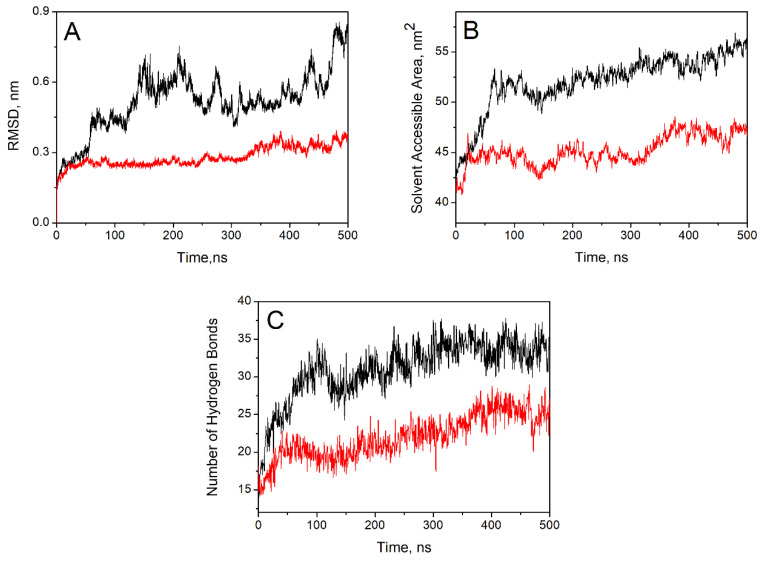
Dependence of MD-derived parameters on the length of simulation time for mixtures of DMSO with BuMeImAcO (black curves) and AlBzMe**_2_**NAcO (red curves). Part (**A**) shows the root mean square deviation (RMSD) curves. Part (**B**) shows the variation of SASA of the SF crystal during the simulations. Part (**C**) shows the number of hydrogen bonds between the SF crystal (acting as donor) and acetate ions, acting as receptors.

We simulated the dissolution of SF in two representative binary solvent mixtures, namely, BuMeImAcO-DMSO (hereafter designated as IL-1) and AlBzMe**_2_**NAcO-DMSO (hereafter designated as IL-2). The arrow inserted into the RDF curves of Figure S2 shows that the extension of the first solvation layer of the SF crystal is the same for both binary mixtures, namely 0.367 nm. Consequently, it is safe to explain the results of IL-1 and IL-2 based on different SF–solvent interactions due to differences in the molecular structure of the ILs. Figure 7 shows MD-based curves, including RMSD (part A), SASA of the SF crystal (part B), and the number of H-bonds between SF (H-bond donor) and the acetate ion (H-bond acceptor).

All parts of Figure 7 show markedly different results for IL-1 and IL-2. Thus, the RMSD curves of Figure 7A indicate a larger displacement of SF crystal atoms from their starting positions, reaching 0.80 nm after 500 ns, for IL-1 (black curve). After a slight initial increase, the RMSD curve for IL-2 (red curve) remains fairly constant at ca. 0.26 nm, reaching ca. 0.36 nm after 500 ns simulation time. The greater perturbation of the SF crystal with IL-1 is also reflected in the calculated larger biopolymer SASA (Figure 7B) and number of SF–acetate H-bonds (Figure 7C). Figure S3 shows the RDF curves for IL-1 and IL-2. The limits of the first solvation layers (i.e., the second “dip” of the curve) are 0.754 and 0.848 nm, respectively. Area integration of these layers indicates that there are 4.1 and 4.5 acetate ions solvating each IL cation. Therefore, there are more free acetate ions (in IL-1, when compared with IL-2) available to form H bonds with the donor groups present in the SF. Additionally, AlBzMe_2_N^+^ is bulkier than BuMeIm^+^, 0.199 nm^3^ and 0.276 nm^3^/ion, respectively. Consequently, there are less ions in the solvation layer of IL-2 than in IL-1. In other words, the “local” concentrations of the binary solvent components are expected to be different, as shown in Table 5 for the averaged values calculated during the simulation time, as shown in Figure S4. In summary, the efficiency of IL-1 relative to IL-2 is due, in part, to differences in AcO-SF H-bonding, which is more efficient for the former. As in case of Cel, the rigidity of the phenyl ring of AlBzMe**_2_**NAcO may adversely affect the efficiency of IL-2 [12].

**Figure 8:**
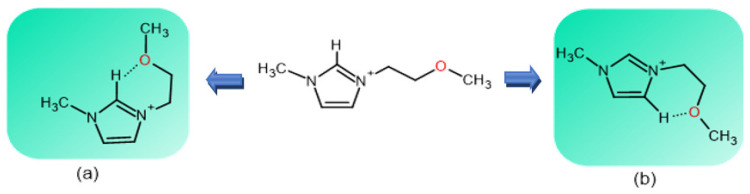
Schematic representation of the MD-based intramolecular hydrogen bonding in 1-(2-methoxyethyl)-3-methylimidazolium acetate, between the ether oxygen, C2-***H***, part (**a**), and C4-***H*** part (**b**) of the 1,3-disubstituted imidazolium ion. This hydrogen bonding “deactivates” the ether oxygen, which is a Lewis base. Figure reproduced with permission from [53]; Elsevier, 2019.

Except for one result, BuMeImAcO-DMSO is more efficient as SF solvent than C_3_OMeImAcO-DMSO, although the latter has an ether linkage that can accept the H-bond, e.g., from -N***H***-CO of SF. This result is in agreement with that observed for the dissolution of Cel in the same binary mixtures. Hence, a similar rational can be advanced: the expected (positive) effect of the ether linkage on SF dissolution is not operative because of the formation of intramolecular H-bonds in the IL proper, as shown in Figure 8 [53]. This explanation also agrees with the fact that C_3_OMeImAcO is a weaker Lewis base than BuMeImAcO, either pure or in mixtures with water [27].

## 4. Conclusions

There is a lack of information in the literature regarding the dissolution of SF; this gap should be addressed. Hence, we studied the dissolution of SF in binary mixtures of DMSO with four ILs pertaining to two chemical classes as a function of *two independent variables*, namely *T* (40, 60, 80 °C) and *χ*_DMSO_ (0.5, 0.7, 0.9). We used a robust experimental dissolution protocol and microscopy for judging SF dissolution. Using chemometrics, we calculated second-order polynomials that correlate SF-m% with both experimental variables. In order to explain the efficiency of the imidazole-based ILs, relative to their quaternary ammonium ion counterparts, we employed MD simulations. We compared the dissolution of a model SF crystal in mixtures of DMSO with BuMeImAcO (efficient solvent; IL-1) and AlBzMe_2_NAcO (less efficient solvent; IL-2). Our MD results showed that SF interacts more strongly with IL-1. This was corroborated by the different concentrations of the solvent components in the solvation layers of the dissolved biopolymer. We checked the robustness of the statistical model by calculating the values of SF-m% under conditions other than those employed to generate the mathematical equations reported in Table 3. The excellent agreement between both values (differences = 3.8 ± 2%) shows the robustness of the statistical model. This satisfying result means that we can predict with confidence the expected values of SF-m%, thus saving time, labor, and material.

## Figures and Tables

**Table 1:** Concentrations of the species in the molecular dynamics’ simulation boxes, calculated and experimental densities.

Ionic Liquid	Species Concentration, mole/L			Solution Density		Δ Density % ^a,b^
	SF	IL	DMSO	Experimental	MD-Based	
BuMeImAcO	3.63 × 10^−3^	2.72	6.36	1.0508	1.0624	1.1
AlBzMe_2_NAcO	3.76 × 10^−3^	2.82	6.58	1.0556	1.0642	0.8

^a^—Density values at 60 °C. ^b^—Δ Density, % = [(MD-Based density − Experimental density)/Experimental density] × 100.

**Table 2:** Dependence of silk fibroin dissolution in ionic liquid/DMSO binary mixtures on the molecular structure of the IL, the temperature, and the composition of the binary mixture.

Entry	Experimental Variable		Mass % of Dissolved Silk Fibroin, SF-m% ^a^			
	Temperature, °C	*χ*_DMSO_^b^	BuMeImAcO ^c^	C_3_OMeImAcO ^c^	AlBzMe_2_AcO ^c^	Bu_4_NAcO ^c^
1	40	0.5	3.0	2.0	0.8	0.3
2	40	0.7	4.0	3.0	1.0	1.0
3	40	0.9	2.0	1.0	0.7	0.5
4	60	0.5	10.0	7.4	0.9	4.0
5	60	0.7	11.0	8.0	3.0	6.0
6	60	0.7	11.5	9.9	3.5	5.0
7	60	0.7	11.5	10.0	3.5	5.0
8	60	0.7	11.5	8.0	3.5	5.9
9	60	0.9	5.5	5.0	2.0	4.8
10	80	0.5	11.0	13.3	7.0	14.0
11	80	0.7	12.0	14.4	8.0	16.0
12	80	0.9	7.0	6.6	4.0	11.0

^a^—Mass percent of dissolved silk fibroin = [mass dissolved SF/(mass dissolved SF + mass IL + mass DMSO)] × 100. Based on the results of the central points for which we have more data points, we calculated the uncertainty in SF-m% from [(SF-m%_maximum value_ − SF-_m%minimum value_)/SF-m%_maximum value_) × 100. Eliminating the (single) worst offender for each IL, we have the following uncertainties: 4.3, 20.0; 14.3, and 16.7% for BuMeImAcO, C_3_OMeImAcO, AlBzMe_2_NAcO, and Bu_4_NAcO, respectively. ^b^—*χ*_DMSO_ = Concentration of dimethyl sulfoxide in the binary solvent on in the mole fraction scale. ^c^—Abbreviations: BuMeIm AcO = 1-(*n*-butyl)-3-methyimidazolium acetate; C**_3_**OMeIm AcO = 1 1-(2-methoxyethyl)-3-methylimidazolium acetate; Bu_4_NAcO = tetra(*n*-butyl)ammonium acetate; AlBzMe**_2_**AcO = allylbenzyldimethyl ammonium acetate.

**Table 3:** Regression equations for the dependence of the mass% of dissolved silk fibroin (SF-m%) on the dissolution temperature (*T*) and the mole fraction of DMSO in the binary solvent, *χ*_DMSO_, *using reduced variable values,* and a second-order polynomial fit.

Entry	IL	Regression Equation	R^2^
1	BuMeImAcO	SF-m% = 2.85 + 19.25*T* − 10.75*T*^2^ + 10.08(*χ*_DMSO_) − 11.75(*χ*_DMSO_)^2^ − 3.0 *T*·(*χ*_DMSO_)	0.978
2	C_3_OMeImAcO	SF-m% = 1.30 + 13.78*T* − 1.50*T*^2^ + 10.98(*χ*_DMSO_) − 11.50(*χ*_DMSO_)^2^ − 5.70*T*·(*χ*_DMSO_)	0.962
3	AlBzMe_2_NAcO	SF-m% = −0.11 + 1.35*T* + 5.60*T*^2^ + 7.38(*χ*_DMSO_) − 6.60(*χ*_DMSO_)^2^ − 2.90*T*·(*χ*_DMSO_)	0.929
4	Bu_4_NAcO	SF-m% = −0.39 + 4.52*T* + 10.15*T*^2^ + 7.18(*χ*_DMSO_) − 6.25(*χ*_DMSO_)^2^ − 3.20 *T*·(*χ*_DMSO_)	0.979

R^2^ is the regression correlation coefficient.

**Table 4:** Comparison of calculated and experimental values under conditions different from those employed to generate the mathematical model.

Entry	Ionic Liquid	Variables Employed	(SF-m%) Calculated	(SF-m%) Experimental	∆SF-m%
1	BuMeImAcO	50 °C/0.6 (DMSO)	9.6	10	4.0
2	BuMeImAcO	70 °C/0.8 (DMSO)	10.5	10.1	−4.0
3	C_3_OMeImAcO	50 °C/0.8 (DMSO)	10.8	11	1.8
4	C_3_OMeImAcO	70 °C/0.6 (DMSO)	13.5	13.9	2.9
5	Bu_4_NAcO	70 °C/0.6 (DMSO)	10.3	9.7	−5.8
6	AlBzMe_2_AcO	70 °C/0.8 (DMSO)	5.1	5.0	−2.0

∆SF-m% = [(Experimental value − calculated value/experimental value)] × 100.

**Table 5:** Averaged number of chemical species (IL^+^, AcO^−^, and DMSO) inside the first solvation shell of SF crystal for both simulations ^a^.

IL	IL Cation	Acetate Ion	DMSO Molecules
BuMeImAcO	48 (0.29)	32 (0.19)	88 (0.52)
AlBzMe_2_NAcO	40 (0.28)	25 (0.18)	77 (0.54)

^a^—The average number of species is within the first solvation layer of SF located at 0.367 nm, as shown in Figure S2. The numbers within brackets refer to the composition of the solvation layer, which is expressed in the mole fraction scale.

## Data Availability

Data is contained within the article and Supplementary Materials.

## Supplementary Materials

The following are available online at https://www.mdpi.com/article/10.3390/polym14010013/s1, Figure S1: The molecular structures of the parallel and antiparallel SF chains employed in the MD simulations; Figure S2: Calculated extension of the first solvation layer of the SF crystal in different IL/DMSO; Figure S3: The RDF curves for IL-1 and IL-2; Figure S4: Local concentrations of the binary solvent components as a function of simulation time; Table S1: Randomized order of carrying out the SF dissolution experiments for each IL-DMSO binary solvent; Table S2: Second-order polynomial fit of the dissolution data; Table S3: Dependence of ET(WB) on T at a fixed χDMSO and on χDMSO at a fixed T.
