# Supplementary material for: Dissolution of Silk Fibroin in Mixtures of Ionic Liquids and Dimethyl Sulfoxide: On the Relative Importance of Temperature and Binary Solvent Composition
Source: Polymers (Basel). 2021 Dec 21;14(1):13. doi: 10.3390/polym14010013 (PMC8747519; doi:10.3390/polym14010013)
Supplement: Supplementary file 1 [file polymers-14-00013-s001.zip › polymers-1385719-supplementary.pdf]

Supporting Material for

Dissolution of Silk Fibroin in Binary Mixtures of Ionic Liquids and Dimethyl Sulfoxide: Assessment of the Relative Importance of Temperature and Binary Solvent Composition

Omar A. El Seoud,[1,*] Marc Kostag,[1] Shirley Possidonio,[2] Marcella T. Dignani,[1] Paulo A. R. Pires,[1] Matheus C. Lourenço.[1]

1-Institute of Chemistry, the University of São Paulo, 05508-000 São Paulo, SP, Brazil,

e-mail: elseoud.usp@gmail.com

2- Department of Chemistry, Institute of Environmental, Chemical, and Pharmaceutical Sciences, University Federal of São Paulo, São Paulo, Brazil

Table S1 Randomized order of the silk fibroin dissolution experiments, using factorial 3^2 planning. [a]

Entry	Designation for T[(b)]	Designation for χ_{DMSO}[(c)]
**1**	-1	-1
**2**	0	-1
**3**	1	-1
**4**	-1	0
**5**[(d)]	0	0
**6**[(d)]	0	0
**7**[(d)]	0	0
**8**[(d)]	0	0
**9**	1	0
**10**	-1	1
**11**	0	1
**12**	1	1

(a) We used the Statistica software to generate this randomized experimental order.

(b) -Designation for the dissolution temperature of SF. The numbers -1, 0 and 1 refer to the experiments carried out at 40, 60, 80 °C, respectively.

(c)- Designation for the mole fraction of DMSO in the binary solvent mixture. The numbers -1, 0 and 1 refer to the experiments carried out at χ_{DMSO} = 0.5, 0.7 and 0.9, respectively.

(d)- Central point, i.e., the dissolution experiment carried out at t = 60 °C and χ_{DMSO} = 0.7.

Table S2: Regression equations for the dependence of the mass% of dissolved silk fibroin (SF-m%) on the dissolution temperature *T* and the mole fraction of DMSO in the binary solvent, χ_{DMSO}, using *raw experimental data* and a second-order polynomial fit.

**IL**		**R^2**
BuMeImAcO	SF-m% = -61,86 +1.11 T -0.007 T^2 +106.15(χ_{DMSO}) -73.43(χ_{DMSO})^2 -0.19 Tx(χ_{DMSO})	0.978
C_3OMeImAcO	SF-m% = -52.80 +0.60T -0.001T^2 +113.58(χ_{DMSO}) -71.87(χ_{DMSO})^2 -0.36Tx(χ_{DMSO})	0.962
AlBzMe$_2$NAcO	SF-m%= -17.5 -0.30T +0.006T^2 +65.02(χ_{DMSO}) -39.06(χ_{DMSO})^2 -0.20Tx(χ_{DMSO})	0.930
Bu$_4$NAcO	SF-m%= -19.02 -0.16T +0.004T^2 +66.96(χ_{DMSO}) -41.25(χ_{DMSO})^2 -0.18 Tx(χ_{DMSO})	0.978

Table S3 Equations for the dependence of E_T(WB) on *T* and χ_{DMSO}.

Dependence of E_T(WB) on T and ($_{DMSO}$)			
Variable Employed	Ionic Liquid	Regression equation	R^2
Fixed T (60 ºC) and variable χ_{DMSO}	AlBzMe$_2$NAco	E_T(WB)= +56.9 -0.75(χ_{DMSO}) -1.25(χ_{DMSO})^2	1
Fixed χ_{DMSO} (0.6) and variable T	AlBzMe$_2$NAco	E_T(WB) +57.2 +0.01T -0.0005T^2	1
Fixed T (60 ºC) and variable χ_{DMSO}	C_3OMeImAcO	E_T(WB)= +58.1 +2.75(χ_{DMSO}) -6.25(χ_{DMSO})^2	1
Fixed χ_{DMSO} (0.6) and variable T	C_3OMeImAcO	E_T(WB) +57.5 +0.11T -0.0019 T^2	1

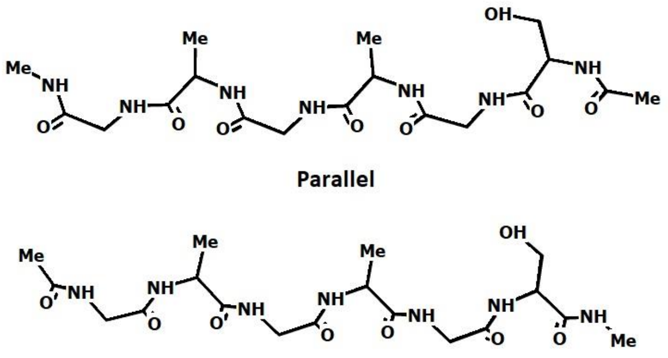

Figure S1: Molecular structures of the model for the parallel and antiparallel silk fibroin chains employed in molecular dynamics calculations.

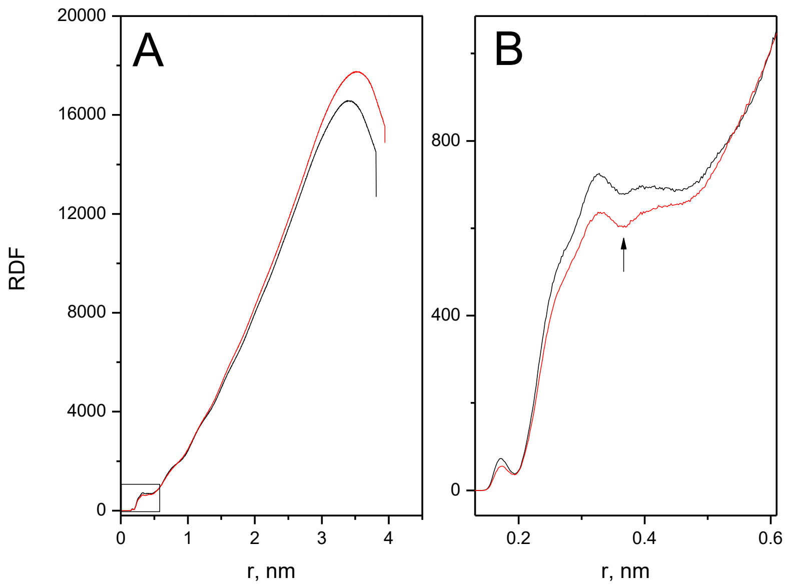

Figure S2 Molecular dynamics-generated radial distribution function plots, for the dissolution of silk fibroin in mixtures of DMSO with BuMeImAcO (black curve) and $AlBzMe_2NAcO$ (red curve). In part B we amplified the square shown in part A. The arrow in part B shows the limit of the first solvation layer of SF crystal (0.367 nm, for both simulations).

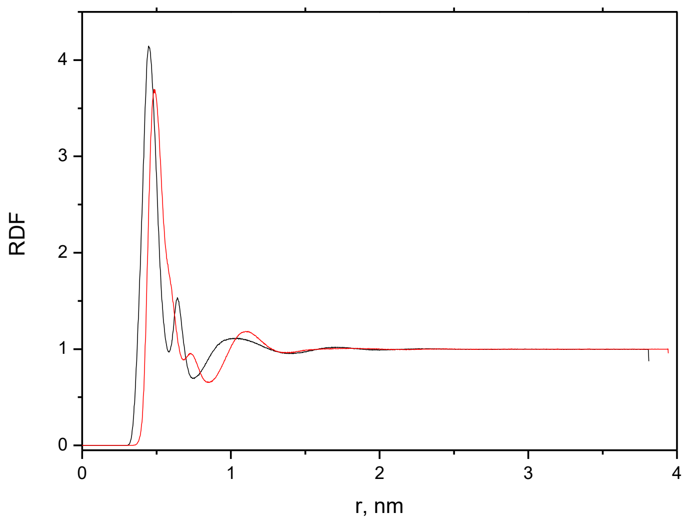

Figure S3. RDF between the center of mass (COM) of acetate and the COM of $BuMeIm^+$ (black curve) or $AlBzMe_2N^+$ (red curve).

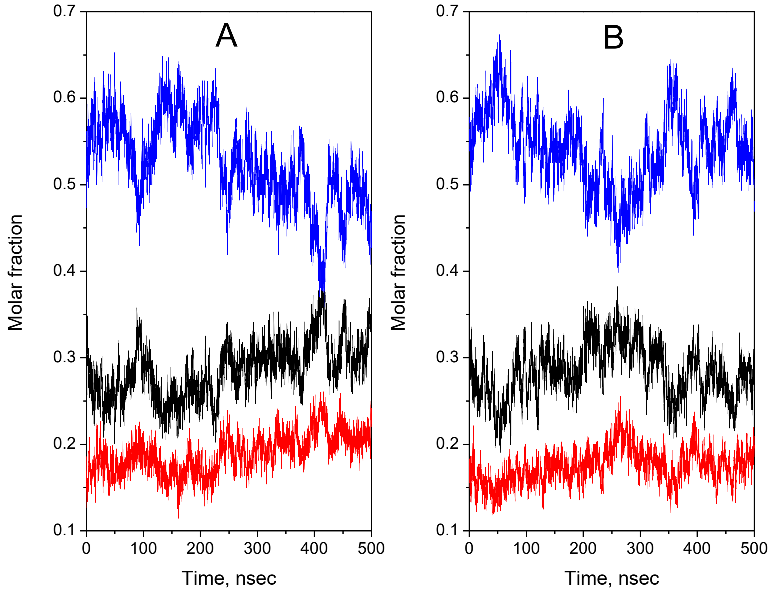

Figure S4. Variation of the composition (in mole fraction) of solvent components in the first solvation layer of SF crystal as a function of simulation time in BuMeImAcO/DMSO (part A) and in $AlBzMe_2NAcO$/DMSO (part B). The colors of the curves are: black, $BuMeIm^+$ or $AlBzMe_2N^+$; red, acetate ion; blue, DMSO.
